# Predictive factors of atrial fibrillation after coronary artery bypass grafting

**DOI:** 10.1590/S1679-45082016AO3673

**Published:** 2016

**Authors:** Cynthia de Oliveira Folla, Cinthia Cristina de Santana Melo, Rita de Cassia Gengo e Silva

**Affiliations:** 1 Instituto do Coração, Hospital das Clínicas, Faculdade de Medicina, Universidade de São Paulo, São Paulo, SP, Brazil.; 2 Escola de Enfermagem, Universidade de São Paulo, São Paulo, SP, Brazil.

**Keywords:** Atrial fibrillation, Arrhythmias, cardiac, Myocardial revascularization, Cardiac surgical procedures, Cardiovascular nursing, Critical care

## Abstract

**Objective:**

To analyze predictive demographic and perioperative variables of postoperative atrial fibrillation in patients who underwent exclusively coronary artery bypass grafting.

**Methods:**

This was a retrospective cohort. We randomly selected 105 medical records of patients who underwent exclusively coronary artery bypass grafting in 2014. Demographic, clinical (preoperative and immediate postoperative) data and related with surgical procedure were collected from medical records. The occurrence of postoperative atrial fibrillation was considered until the third day after the surgery. Variables were analyzed using descriptive and inferential statistics. To identify predictive factors of postoperative atrial fibrillation we used a decision tree model with Classification and Regression Trees algorithm.

**Results:**

Atrial fibrillation incidence was 19.0% (n=20). Patients with left atrial >40.5mm and aged >64.5 years were more likely to develop the arrhythmia during the post-surgical period.

**Conclusion:**

Left atrial diameter and advanced age were predictive factors of atrial fibrillation in patients who underwent exclusively coronary artery bypass grafting.

## RESUMO

**Objetivo:**

Analisar as variáveis demográficas e perioperatórias preditivas de fibrilação atrial pós-operatória em pacientes brasileiros submetidos exclusivamente à cirurgia de revascularização do miocárdio.

**Métodos:**

Trata-se de coorte retrospectiva. A amostra foi constituída de 105 prontuários de pacientes submetidos exclusivamente à revascularização do miocárdio no ano de 2014, selecionados aleatoriamente. Dados demográficos, clínicos (préoperatórios e do pós-operatório imediato) e relacionados ao procedimento cirúrgico foram coletados por meio de consulta ao prontuário. A ocorrência de fibrilação atrial no pós-operatório foi considerada até o terceiro dia após a cirurgia. As variáveis foram analisadas por estatística descritiva e inferencial. Para identificar os fatores preditivos de fibrilação atrial no pós-operatório, utilizou-se um modelo de árvore de decisão com algoritmo *Classification and Regression Trees*.

**Resultados:**

A incidência de fibrilação atrial pós-operatória foi de 19,0% (n=20). Pacientes com átrio esquerdo >40,5mm e idade >64,5 anos foram aqueles com mais chance de desenvolver a arritmia no pós-operatório.

**Conclusão:**

Tamanho do átrio esquerdo e idade avançada foram fatores preditivos de fibrilação atrial em pacientes submetidos exclusivamente à cirurgia de revascularização do miocárdio.

## INTRODUCTION

Atrial fibrillation (AF) is a supraventricular arrhythmia characterized by chaotic atrial activity and high frequency, which electrocardiographic record shows *f* waves in the baseline that varying in shape and amplitude.^(1)^ This is one of the most common arrhythmia after heart surgery which incidence peak occurs until the third day in the postoperative period.^(2)^

Studies show that incidence of postoperative atrial fibrillation (POAF) in patients who underwent heart surgery varies from 10 to 60%. Literature suggests that patients undergoing exclusively coronary artery bypass grafting (CABG) has lower incidence of POAF (15 to 40%) compared with those who undergone this surgery combined with valve replacement (62%).^(3)^ Although the type of surgery can influence the occurrence of POAF, we did not observe difference in incidence of arrhythmia in patients who underwent surgery with or without extracorporeal circulation (ECC).^(3,4)^

Until recently, the POAF was recognized as benign complication. However, a systematic review with meta-analysis showed that this arrhythmia is associated to higher mortality rates at short and long term. Patients who developed POAF had higher prevalence of complications after surgery such as stoke, pneumonia, respiratory failure, and longer hospitalization.^(5)^

Recognizing factors associated with arrhythmia is paramount to reinforce surveillance and monitor patients at risk, as well as to implement prophylactic measures to avoid deleterious effects of POAF. Predictive models has been tested to estimate the risk of POAF development after heart surgery.^(6-8)^ A number of models were proposed based only in pre, intra or postoperative data, and using predictive models of stroke in patients with AF to predict POAF or by analyzing patients who underwent combined heart surgery (coronary artery bypass grafting and valve replacement).^(9-11)^ In addition, Brazilian studies evaluating predictive factors to POAF are scarce. Therefore, it is important to recognize the contribution of perioperative variables and analyze them in groups, in order to develop POAF in Brazilian patient who underwent exclusively CABG.

## OBJECTIVE

To evaluate predictive demographic and perioperative variables for atrial fibrillation after the surgery in Brazilian patients who underwent exclusively coronary artery bypass grafting.

## METHODS

This was retrospective cohort study was carried out at a single Brazilian center. Our study was approved by the Ethical and Research Committee from the institution that proposed the investigation (number 995,451, CAAE 42471715.7.0000.5392). Researches followed all ethical recommendations. The consent form was waived because this is a retrospective study.

We included 105 medical records, selected randomly by drawing. The sample was calculated considering prevalence of POAF in CABG and mean number of surgeries carried out monthly in the analyzed period. To be included in the drawing, medical records need to belong to Brazilian patients aged 18 years or older, of both sexes, and who underwent exclusively CABG between January to July 2014 at a public hospital specialized in cardiology in the city of São Paulo (SP). We excluded medical records of patients diagnosed with pre-operative AF.

The drawing was carried out using monthly surgery lists from the analyzed period. Patients with indication to undergone isolated GABG were identified and they received a coding number. Forms including same coding numbers were inserted into an envelope for the drawing. In order to avoid seasonality, a total of 15 medical records were selected every month.

Preoperative variables analyzed were: age, sex, comorbidities, medications used at home, results of laboratorial exams and echocardiogram. Variables related to surgical procedures were: type of surgery (elective or emergency) use of ECC, time of surgery and amount of grafts. In immediate postoperative we obtained results of laboratorial exams and use of vasoactive drugs. The occurrence of POAF was evaluated since immediate postoperative until the third day after surgery, because this period had a higher incidence of arrhythmia.^(2)^ The diagnosis of POAF was considered when a written diagnosed existed in the medical record concerning the new episode of AF, *i.e*., which occurred after the surgery, also including the institution in which the therapeutic measure for arrhythmia was also present.

Descriptive and inferential statistics were used for data analysis. To compare groups with and without POAF related with categorical variables we used Fisher’s exact test and χ^2^ test. To compare means the Mann-Whitney test was applied after the Kolmogorov-Smirnov normality test. Predictive factors of POAF were identified using the decision tree algorithm, Classification and Regression Trees (CART), in which all perioperative variables were included. The model was adjusted with all variables to predict AF. In each knot of the tree, variables were verified, to determine cohort cut-offs that make groups more equal POAF. In case of lacking values in the separation variable, the second best predictor was used as substitute variable in those cases. We used a cross validation to enable generalization of results from population tree. To increase sensibility of model, the cost of misclassification of two was used for AF cases, *i.e*., for regression tree estimations, we considered that it would be twice as worse to classify patients with POAF as without POAF than otherwise. Complexity parameter was used as criterion of stopping the decision tree. We calculated values of sensibility, specificity and precision of the tree. The level of significance adopted to all tests was 5%.

## RESULTS

The incidence of POAF was 19.0% (n=20). The table 1 shows comparison of patients with and without POAF in relation to demographic and clinical variables analyzed before the surgery.

**Table 1:** Demographic and clinics characteristics before the surgery described in analyzed medical records

Variables	With POAF (n=20)	Without POAF (n=85)	p value
Age, years	67.7 (7.4)	62.1 (9.4)	0.013*
Male	17 (85.0)	64 (75.3)	0.554†
White	19 (95.0)	73 (85.9)	0.454†
Blood hypertension	15 (75.0)	77 (90.6)	0.123†
Dyslipidemia	15 (75.0)	64 (75.3)	1.000†
*Diabetes mellitus*	5 (25.0)	44 (51.8)	0.031‡
Previous myocardial infarction	4 (20.0)	25 (29.4)	0.397‡
Smoking	3 (15.0)	13 (15.3)	1.000†
Heart failure	3 (15.0)	11 (12.9)	0.738†
Chronic renal diseases	1 (5.0)	6 (7.1)	1.000†
Chronic obstructive pulmonary disease	0 (0.0)	1 (1.2)	1.000†
Left ventricular ejection fraction	58.5 (10.9)	57.7 (11.4)	0.784*
Size of left atrium (mm)	41.8 (4.8)	38.8 (5.2)	0.020*
Mass index (g/m^2^)	99.9 (26.3)	97.4 (23.6)	0.567*

*Mann-Whitney test; Fisher’s exact test; ‡χ^2^ test. Results presented in means (standard deviation).

POAF: postoperative atrial fibrillation.

Patients who developed POAF were older and had lower prevalence of *diabetes mellitus*. The ejection fraction of left ventricle was within normal limits for patients in both groups, however, the size of left atrium was significantly larger in those who developed arrhythmia after the surgery.

Statins was prescribed to all patients without and with POAF (81.2% *versus* 70.0%, respectively, p=0.358), in addition to angiotensin-converting enzyme inhibitors (64.7% *versus* 45.0%; p=0.104) and antiarrhythmic agent (65.9 *versus* 60.0%; p=0.620). Results of laboratorial exams before the surgery did not show difference among patients with or without POAF (Table 2).

**Table 2:** Results of laboratorial exams before the surgery described in medical records

Variables	With POAF (n=20)	Without POAF (n=85)	p value*
Magnesium, mEq/L	1.8 (0.2)	1.7 (0.2)	0.913
Sodium, mEq/L	137.7 (3.8)	139.1 (3.1)	0.099
Potassium, mEq/L	4.32 (0.5)	4.3 (0.4)	0.914
Calcium, mg/dL	1.2 (0.1)	1.3 (0.6)	0.351
CRP, mg/dL	12.9 (19.7)	9.0 (13.5)	0.129
Creatinine, mg/dL	1.4 (1.4)	1.6 (1.9)	0.645
Urea, mg/dL	44.9 (30.4)	44.0 (24.3)	0.819

*Mann-Whitney test. Results presented in means (standard deviation).

POAF: postoperative atrial fibrillation; CRP: C-reactive protein.

The majority of surgeries were elective (n=95; 90.4%). We did not observe statistical difference between groups with and without POAF concerning the type of surgery (elective surgery: 92.9% *versus* 80.0%; p=0.094) and mean number of grafts performed (2.7±0.7 *versus* 2.5±1.0; p=0.194). No difference between groups with and without POAF was seen in the use or not of ECC (60.0% *versus* 65.8%; p=0.620), time of ECC (47.2±41.7 minutes *versus* 58.6±47.8 minutes; p=0.289), time of anoxia (58.8±14.8 minutes *versus* 65.2±65.2 minutes, p=0.447) and total duration of the surgery (416.2±69.2 minutes *versus* 435.6±92.5 minutes; p=0.543).

In the immediate postoperative, all patients were using, at least, one vasoactive drug, predominantly dobutamine in both groups. Results of laboratorial tests are described in table 3.

**Table 3:** Minimal, medium and maximal values of laboratorial tests results in the immediate postoperative

Variables	Minimal	p value*	Maximal	p value*	Mean	p value*
Magnesium, mEq/L						
With POAF	1.5 (0.2)	0.079	1.9 (0.3)	0.170	1.7 (0.2)	0.051
Without POAF	1.5 (0.3)		1.8 (0.3)		1.8 (0.3)	
Sodium, mEq/L						
With POAF	135.6 (3.3)	0.931	138.7 (3.2)	0.343	137.1 (3.0)	0.483
Without POAF	132.7 (19.8)		139.3 (3.5)		136.8 (5.8)	
Potassium, mEq/L						
With POAF	3.8 (0.4)	0.783	4.5 (0.4)	0.563	4.1 (0.3)	0.903
Without POAF	3.8 (0.4)		4.5 (0.6)		4.1 (0.5)	
Ionized calcium, mg/dL						
With POAF	1.12 (0.03)	0.018	1.21 (0.08)	0.467	1.16 (0.04)	0.245
Without POAF	1.14 (0.05)		1.22 (0.08)		1.18 (0.06)	
CRP, mg/dL						
With POAF	57.8 (73.9)	0.451	65.0 (71.9)	0.761	64.1 (73.0)	0.680
Without POAF	39.7 (31.0)		54.8 (32.1)		47.0 (26.9)	
Creatinine, mg/dL						
With POAF	2.5 (3.0)	0.074	2.2 (2.7)	0.161	2.2 (2.7)	0.129
Without POAF	1.7 (2.2)		2.3 (4.9)		2.0 (3.1)	
Urea, mg/dL						
With POAF	45.6 (38.5)	0.699	46.1 (38.4)	0.537	45.8 (38.5)	0.613
Without POAF	46.4 (28.5)		47.8 (29.8)		47.1 (29.1)	

*Mann-Whitney test. Results presented in means (standard deviation).

POAF: postoperative atrial fibrillation; CRP: C-reactive protein.

Although serum electrolytes were within normal limits, patients who developed POAF had minimal value of serum calcium significantly lower than those who did not develop POAF.

All patients had positive hybrid balance immediate after surgery and no difference between groups concerning this variable was seen (2,691.6±724.5mL *versus* 2,404.2±1; 293.9mL for patients with or without POAF; p=0.328).

**Figure 1:**
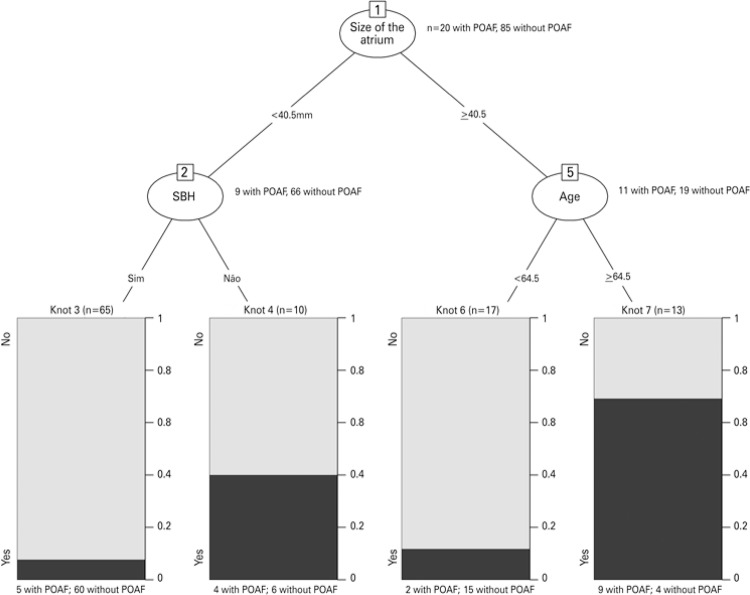
Predictive factors for postoperative atrial fibrillation (POAF) of coronary artery bypass grafting. Knot 1: size of left atrium (40.5mm) was the best variable to identify patient who developed POAF. Knot 2: the diagnosis of arterial hypertension identified patients with and without POAF when the size of the left atrium was smaller than 40.5mm. Knot 5: age identified patients who developed POAF when size of left atrium was >40.5mm

SBH: Systemic blood hypertension; POAF: postoperative atrial fibrillation.

Figure 1 shows predictive variables of POAF in the sample. We observed that size of left atrium varied and it better identified patients without POAF. According to decision tree, patients with left atrium ≥40.5mm and aged ≥64.5 years had more chance to develop arrhythmia after the surgery. Sensibility of this prediction model was 65% and specificity, 88.2%, predictive positive and negative values were 56.5% and 91.5%, and the precision was 83.8%.

## DISCUSSION

This study enabled to explore contribution of perioperative variables in prediction of POAF in Brazilian patients submitted exclusively to CABG. In addition, because these are surgical procedures done in the last years, these results considered the use of current surgical techniques, which could influence outcomes of interest. Although the sample analyzed would be small, results presented provided support to advance score proposition that estimate the risk of developing arrhythmia.

The incidence of POAF was 19.0% (n=20) which agrees with data from the literature.^(2-4,12)^ The mean age of patients who developed POAF was significantly higher than in patients who had arrhythmia. The anatomical and functional changes related to ageing and worsening of clinical conditions can contribute for this finding. Advanced age is a factor described in different studies as a risk for development of POAF in heart surgeries.^(3,8,13,14)^

A study that identified predictive preoperative characteristics of POAF and CABG reported that age was the more important isolated predictive of POAF (OR=1,059/year, 95% confidence interval - 95% CI: 1,055-1,063).^(13)^ In another study that evaluated factors that influence occurrence of AF after endoscopic CABG, we verified that age and body weight were the predictive factors.^(14)^

Patients who developed POAF had significantly lower prevalence than diabetes. According to the decision tree, however, the diagnosis of diabetes was not a predictive variable of POAF. However, some studies identified the influence of diabetes in AF incidence, but results still controversial.^(15,16)^

We did not observe association of other demographic variables and comorbidities with the development of POAF. Differently, results of the Framingham’s study suggests an association between sex, blood hypertension and heart failure with development of AF in community patients.^(17)^ Still, there are evidences that white people have independent risk factor for POAF in isolated CABG.^(18)^

In relation to preoperative echocardiographic data, we observed that patients who developed arrhythmia after CABG had larger left atrium than those who developed POAF. In addition, the size of the left atrium was the variable that best identified patients who developed POAF. The physiopathological mechanisms of POAF are not fully understood. We understand, however, that structural factors of the heart seem to be related to higher vulnerability for the development of arrhythmia after the surgery, such as increase of size of atrium.^(3)^ Medical literature shows that patients who developed AF after CABG had higher atrial area compared with those who developed arrhythmia.^(19)^

No association of intraoperative variables was seen with development of POAF. Other authors had reported similar results concerning the type of surgery.^(8)^ In relation to use of ECC, researchers concluded that CABG without ECC did not reduce the incidence of POAF.^(4)^ Measures that reflect intraoperative time of ischemic heart, such as aortic clamp time, have been reported as risk factors for POAF, although literature still controversial on the subject.^(3,20)^

Electrolytic imbalance has been associated with occurrence of POAF in CABG.^(3)^ In our study, lower serum levels of calcium immediate after surgery were associated with development of AF, but this not distinguished, in tree decision model, patients with and without POAF. In fact, such result should be confirmed in future studies because evidences suggest that calcium overload from the reperfusion of ischemic areas is one of the underlying arrhythmogenic mechanisms of arrhythmias after the heart surgeries.^(21)^ In addition, the use of blocking calcium channels in prophylaxis of POAF is controversial.^(22,23)^

Although association between CRC with development of POAF was not observed in our study, it is important to highlight that literature suggests the participation of inflammatory component in the development of POAF in CABG, which is supported by evidences that anti-inflammatory drugs use, including statins, reduces the incidence of arrhythmia.^(3)^

This study had limitations. The retrospective design, the convenient sample and data collection at single center can be identified as compromising factors to extrapolate results. Data collection from medical records can also compromise results because quality of documenting health records is affected by different factors, including its integrality. Another limitation that should be taken into consideration is the definition adopted of POAF, *i.e*., the arrhythmia episode documented in medical record by the physician and those who needed antiarrhythmic agents, we did not considered transient episodes. Observance of time limit until the third day after surgery contributed for limit the number of late cases of POAF, including those developed after hospital discharge. Confounding variables effect should not be ignored, which were not evaluated in our study, such as surgical technique employed, and also type and dosage of vasoactive drugs.

## CONCLUSION

The increase of size of left atrium and advanced aging were predictive factors of atrial fibrillation after the surgery in patients who underwent exclusively coronary artery bypass grafting.
